# CYP17A1 deficient XY mice display susceptibility to atherosclerosis, altered lipidomic profile and atypical sex development

**DOI:** 10.1038/s41598-020-65601-0

**Published:** 2020-05-29

**Authors:** Redouane Aherrahrou, Alexandra E. Kulle, Natalia Alenina, Ralf Werner, Simeon Vens-Cappell, Michael Bader, Heribert Schunkert, Jeanette Erdmann, Zouhair Aherrahrou

**Affiliations:** 1 Institute for Cardiogenetics, University of Lübeck, Lübeck, Germany; 2 Centre for Public Health Genomics, Department of Biomedical Engineering, University of Virginia, Charlottesville, VA USA; 3 Department of Pediatrics, Division of Pediatric Endocrinology and Diabetes, University Hospital Schleswig-Holstein, Christian-Albrechts-University, Kiel, Germany; 4 Max-Delbrück-Center for Molecular Medicine (MDC), Robert-Rössle-Str. 10, 13125 Berlin, Germany; 5 DZHK (German Centre for Cardiovascular Research), Partner Site Berlin, Berlin, Germany; 6 Department of Pediatrics, Division of Pediatric Endocrinology and Diabetes, University of Lübeck, Lübeck, Germany; 7 Institute of Molecular Medicine, University of Lübeck, Lübeck, Germany; 8 Bioanalytical Core Facility, CBBM (Center of Brain Behavior and Metabolism), University of Lübeck, Lübeck, Germany; 9 Charité-Universitätsmedizin, Berlin, Germany; 10 Institute for Biology, University of Lübeck, Lübeck, Germany; 11 Kardiologie, Deutsches Herzzentrum München, Technische Universität München and DZHK (German Centre for Cardiovascular Research), Partner Site Munich Heart Alliance, Munich, Germany; 12 DZHK (German Centre for Cardiovascular Research), Partner Site Hamburg/Kiel/Lübeck, Germany, University Heart Centre Lübeck, 23562 Lübeck, Germany

**Keywords:** Cardiovascular genetics, Endocrinology

## Abstract

CYP17A1 is a cytochrome P450 enzyme with 17-alpha-hydroxylase and C17,20-lyase activities. *CYP17A1* genetic variants are associated with coronary artery disease, myocardial infarction and visceral and subcutaneous fat distribution; however, the underlying pathological mechanisms remain unknown. We aimed to investigate the function of CYP17A1 and its impact on atherosclerosis in mice. At 4–6 months, CYP17A1-deficient mice were viable, with a KO:Het:WT ratio approximating the expected Mendelian ratio of 1:2:1. All *Cyp17a1* knockout (KO) mice were phenotypically female; however, 58% were Y chromosome-positive, resembling the phenotype of human CYP17A1 deficiency, leading to 46,XY differences/disorders of sex development (DSD). Both male and female homozygous KO mice were infertile, due to abnormal genital organs. Plasma steroid analyses revealed a complete lack of testosterone in XY-KO mice and marked accumulation of progesterone in XX-KO mice. Elevated corticosterone levels were observed in both XY and XX KO mice. In addition, *Cyp17a1* heterozygous mice were also backcrossed onto an *Apoe* KO atherogenic background and fed a western-type diet (WTD) to study the effects of CYP17A1 on atherosclerosis. *Cyp17a1* x *Apoe* double KO XY mice developed more atherosclerotic lesions than *Apoe* KO male controls, regardless of diet (standard or WTD). Increased atherosclerosis in CYP17A1 XY KO mice lacking testosterone was associated with altered lipid profiles. In mice, CYP17A1 deficiency interferes with sex differentiation. Our data also demonstrate its key role in lipidomic profile, and as a risk factor in the pathogenesis of atherosclerosis.

## Introduction

Coronary artery disease (CAD) is a major cause of morbidity and mortality worldwide^1^. Inheritance plays an essential role in the pathogenesis of CAD, and recent genome-wide association studies (GWAS) have revealed more than 160 genomic loci associated with this condition^2^. One commonly reported genomic locus, for CAD was located at chr10p24.32. The same locus is also associated with myocardial infarction (MI)^3^ and visceral and subcutaneous fat distribution^4^. This locus harbors the *CYP17A1* gene, which encodes the cytochrome P450 17A1 enzyme, also referred to as steroid 17-alpha-monooxygenase or 17α-hydroxylase/17,20-lyase/17,20-desmolase. Mutations affecting CYP17A1 function are reported to cause congenital adrenal hyperplasia (CAH), a rare inherited disorder that affects both sexes. Approximately 1 in 13,000 to 15,000 children are born with CAH^5^, with mutations in *CYP17A1* accounting for 1% of CAH in most populations^6^. In Brazil, mutations in *CYP17A1* are frequent among patients with CAH, representing the second most common cause of the condition^7^. CYP17A1 is a critical factor in the steroidogenic pathway producing progestins, glucocorticoids, androgens, estrogens, and mineralocorticoids. In addition to adrenal steroid biosynthesis CYP17A1-deficiency also affects gonadal steroid biosynthesis manifesting as disorders/differences of sex development (DSDs), leading to female or ambiguous external genitalia in 46,XY individuals and lack of pubertal development in boys and girls^8,9^.

CYP17A1 is a key microsomal enzyme that converts pregnenolone and progesterone to their major downstream products: dehydroepiandrosterone (DHEA), cortisol, testosterone, and estradiol^10,11^. Published evidence indicates an association between steroid hormone imbalance and traditional risk factors for CAD. Furthermore, estrogens can have a critical role in CAD, depending on sex; they appear to be detrimental in men and protective in women. More specifically, increased risk of CAD and MI is associated with early menopause in women, while high levels of endogenous estrogens are thought to explain the low prevalence of CAD in pre-menopausal women^12–15^. However, in men high levels of estrogens and estrone are associated with increased risk for MI and CAD^16,17^, while lower testosterone levels have been linked to increased risk for CAD^18–20^. Castration of Apoe knockout male mice led to increase atherosclerosis^20^.

While there is strong evidence that CYP17A1 enzyme synthesis is critical in the steroidogenic pathway to produce progestins, mineralocorticoids, glucocorticoids, androgens, and estrogens, the functional role of CYP17A1 in CAD remains unclear, and animal disease models are required to facilitate in-depth understanding of the underlying molecular mechanisms. In this context, we generated CYP17A1-deficient mice and studied the impact of lack of this gene on steroid metabolism, as well as its roles in atherosclerosis and lipid metabolism.

## Materials and methods

### Ethics statement

All animal experiments were approved by the German animal studies committee of Schleswig-Holstein, and animals were maintained and handled according to international guidelines.

### Generation of Cyp17a1 knockout (KO) mice

**Figure 1:**
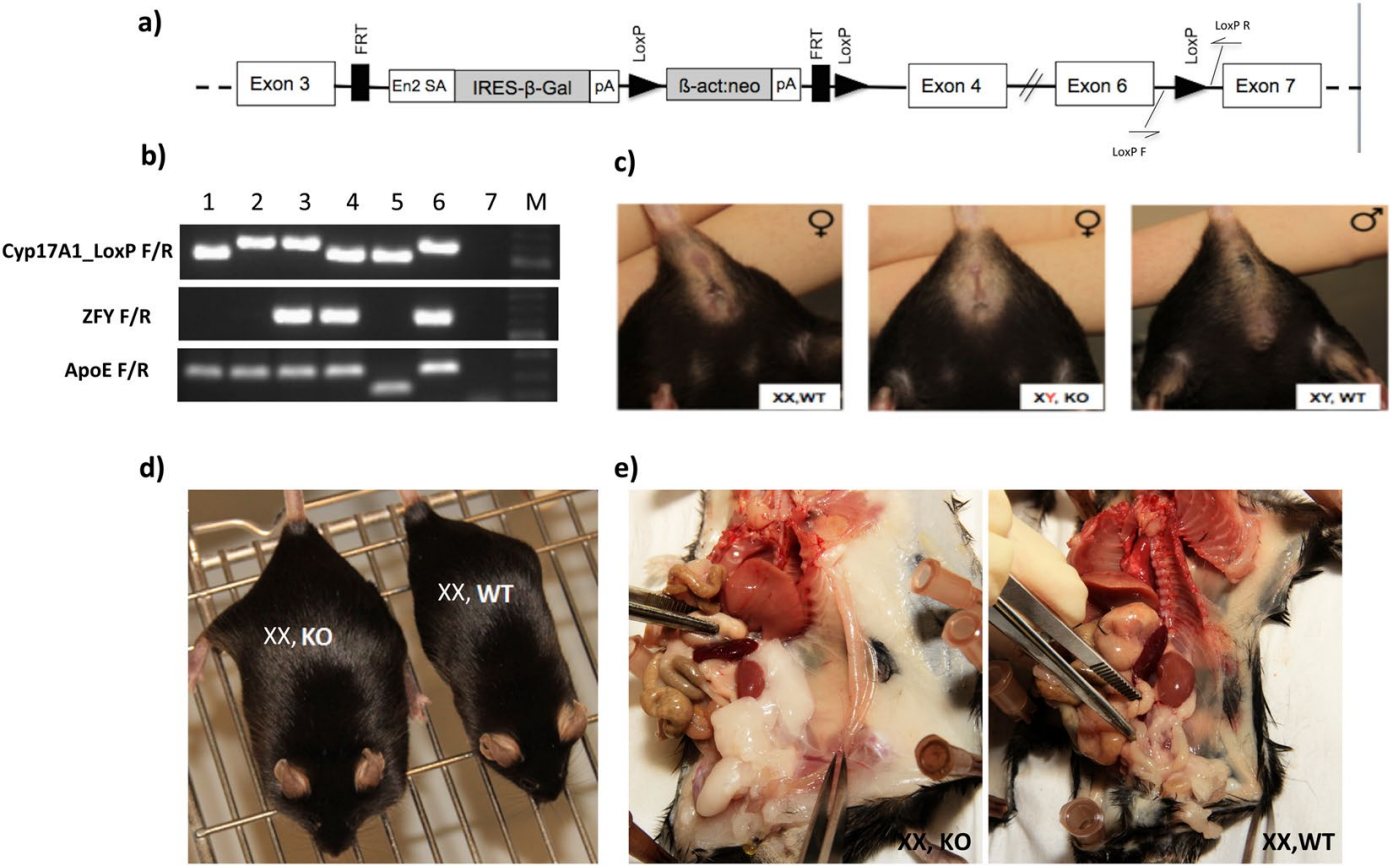
Generation and characteristics of *Cyp17a1* KO mice. (**a**) Targeting vector used to generate *Cyp17a1* KO mice. (**b**) Genotyping of mice by PCR (1: XX, *Cyp17a1*(+/+)*xApoe(d/d)*; 2: *XX,Cyp17a1(d/d)xApoe(d/d)*; 3: *XY,Cyp17a1(d/d)xApoe* (d/d); 4: XY,Cyp17a1(+/+)xApoe(d/d); 5: XX, Cyp17a1(+/+)xApoe(+/+) control; 6: XX, *Cyp17a1(d/d)xApoe(d/d)* control; 7: H_2_O; M: 100 bp marker). (**c**) The female appearance of XY KO mice. (**d**) Increased body weight of an XX KO mouse, compared with a control XX WT littermate. (**e**) Increased visceral fat in an XX KO mouse compared with a control littermate.

The *Cyp17a1* knockout embryonic stem cell (*Cyp17a1*-KO-ESC) line (EPD0214_2_B11) was purchased from the European Conditional Mouse Mutagenesis Program (EUCOMM). After quality control testing, microinjection of *Cyp17a1*-KO-ESC into C57BL/6N blastocysts was achieved. The *Cyp17a1* targeting vector **(**Fig. 1a**)** includes a neomycin selection cassette and two LoxP sites flanking exons 4 and 6 in the *Cyp17a1* genomic sequence. It is also designed as Knockout-first. More specificaly, *Cyp17a* gene expression was interrupted by splicing exon 3 to the ß-galactosidase sequence 3′ of exon 3 (Fig. 1a). The introduced sequence includes En2 *splice acceptor* (En2 SA) and the SV40 polyadenylation sequences (Fig. 1a). Male chimeras were obtained and backcrossed to C57BL/6J females in our animal house to generate heterozygous founders with a C57BL/6J genetic background. As both male and female homozygous knockout mice were infertile, backcrosses were conducted using heterozygous animals. Mice were backcrossed for at least six further generations to generate *Cyp17a1* KO mice on a C57BL/6J background. The following primer pairs were used for the characterization of the knockout mice: Cyp17A1_LoxP F_TCCAGGTAAGCCTTTCTTCC/R_AGAACCCTCCCCCATTCTC flanking the LoxP site in the intronic region between exon 6 and 7; ZFY_F AAGATAAGCTTACATAATCACATGGA/R_CCTATGAAATCCTTTGCTGCACATGT for the detection of the Y chromosome.

### Breeding of the *Cyp17a1* KO onto an atherogenic *Apoe* KO background

C57BL/6J mice [wild type (WT)] and *Apoe(d/d)* atherogenic mice were purchased from Charles River (Germany). To study the role of CYP17A1 in atherosclerosis, *Cyp17a1(*+*/d)* mice were mated with *Apoe(d/d)* animals to generate and select *Cyp17a1(*+*/d)* x *Apoe(d/d)* littermates, which were further bred together to produce *Cyp17a1(d/d)* x *Apoe(d/d)* and *Apoe(d/d)* mice as controls. WT mice (not on the *Apoe(d/d)* background) were used as non-atherogenic controls. Genotyping was conducted as previously described^21^. PCR was used to confirm the disruption of *Apoe*, according to a standard protocol from the Jackson Laboratory.

### Analysis of *Cyp17a1* expression

#### Immunohistochemisty

Embryos, gonadal and aorta tissue had been fixed in 4% paraformaldehyde and embedded in paraffin. Slices were pretreated with sodium citrate buffer (10 mM sodium citrate, 0.05% Tween 20) for epitope demasking and 0.3% hydrogen peroxide to reduce non-specific background due to endogenous peroxidase. CYP17A1 was detected using goat polyclonal antibody sc-46081 (Santa Cruz Biotechnology, Inc) at a dilution of 1:50 and an anti-goat biotinylated secondary antibody (Vectastain BA-5000, Vector Laboratories Inc., Burlingame, CA, USA). Bound antibodies were visualized using the ABC kit (Vectastain, PK-4000) and DAB 2 component chromogen kit (DCS. Hamburg, Germany) or using using donkey anti-goat AP antibody (Supplementary Fig. 2a,b).

#### X-gal staining of cryosections

Organs were excised and embedded in Tissue Tek (Sakura), snap frozen in liquid nitrogen, and stored at −20 °C until use. The X-gal staining was performed as previously reported by our group^22^. Briefly, cryosections of 8–10 μm were air dried and incubated in PBS containing 0.5% glutaraldehyde at 4 °C for 10 min and incubated with X-gal (5-bromo-4-chloro-3-indolyl-β-D-galactopyranoside) solution at 37 °C overnight. Sections were counter-stained with Nuclear Fast Red, dehydrated and mounted using cover slips (Fisher).

#### *qPCR* analysis

The analysis was performed as described previously^21,23^. Briefly, total RNA was isolated from different cultured cells or purchased from commercially available human tissues (Origene) such as Heart (HT1002), Kidney (HT1003), and Liver (HT1005) using the RNeasy kit (Qiagen, Valencia, CA, USA) and reverse transcribed into cDNA. mRNA level of CYP17A1 was determined by relative quantitative RT-PCR and analysed using normalisation to GAPDH. The primers used for gene expression studies are as follow: For human samples analysis huCYP17A1-F/R: (TGGGCACCAAGACTACAGTG/GTTGTTGGACGCGATGTCTA) and huGAPDH-F/R: (GGATTTGGTCGTATTGGG/GGAAGATGGTGATGGGATT) were used. For mice mCyp17a1-F/R (GAGGGTATTGTGGATGTCCTG/GCATTTTTCAAACATTTCAACC) and mGAPDH-F/R (GACCACAGTCCATGCCATCAC/CCGTTCAGCTCTGGGATGAC) were used.

### Analysis of atherosclerosis

#### Induction of atherosclerosis and collection of samples

Atherosclerotic plaque formation was analyzed in both male and female mice with four genotypes, *Cyp17a1(d/d)* x *Apoe(d/d)*, *Apoe(d/d)*, *Cyp17a1(d/d)*, and controls (WT), at 10 weeks old. Each genotype was randomly divided into two groups (n = 6–8), which were fed either standard chow diet (chow) or a western-type diet (WTD), containing 0.2% cholesterol and 21.2% fat (TD.88137, Ssniff Spezialdiäten GmbH, Germany), for an additional 8 weeks. All mice were maintained under controlled conditions: temperature (23 °C), humidity (40–60%), and lighting (12 h/12 h light/dark cycle). Blood was collected from the retro-orbital sinus (following overnight fasting) in EDTA tubes and centrifuged at 4500 x g for 3 min at 4 °C to collect plasma. Plasma samples were collected in the same way before and after differential feeding with the WTD or standard chow. After 8 weeks on the allocated diet, mice were euthanized by inhalation of an overdose of isoflurane, sacrificed by cervical dislocation, and perfused with phosphate-buffered saline (pH 7.4) (Lonza, Cologne, Germany). Aortas (to the iliac bifurcation) and hearts were removed and fixed in 4% paraformaldehyde solution for histological analysis. Additional organs were collected for future studies. Tail biopsies were taken for re-genotyping. All samples for future RNA and protein studies were stored at −80 °C until further analysis.

#### Atherosclerosis analysis

To analyze atherosclerotic lesions in mice, atherosclerotic deposits were quantified in two types of aorta sample: 1) the whole aorta (from the aortic arch to the iliac bifurcation) and 2) the aortic root. All atherosclerotic plaque analyses were performed blind to the mouse genotype.

For the whole aorta, so-called ‘en-face’ analysis was conducted. Briefly, aortas were cleaned of fat and connective tissue, followed by removal of the adventitia. Then, aortas were opened longitudinally to expose the intimal surface and lipid-rich intraluminal lesions. Aortas were stained using Oil Red O (ORO) to detect atherosclerotic deposits. Lesions were quantified from the aortic arch to the iliac bifurcation, including the brachiocephalic trunk, the left common carotid artery, and the left subclavian artery. Images were acquired using Leica equipment and Leica Application Suite (LAS) version 4.2 software (Leica Microsystems, Wetzlar, Germany). After omitting few mice due to bad quality of sectioning 6–8 animals were analyzed by a blinded observer using GIMP software, version 2.6 (The GIMP Development Team), and the ratio of ORO-positive lesions in each animal was determined as the percentage lesion area, normalized to the total area of the aorta.

For quantification of atherosclerosis in the aortic root, including the aortic valves, 10 serial cross-sections (10 μm thickness) were obtained, starting below the aortic root until the proximal aorta, below the aortic arch, using an in-house standardized protocol. The mean atherosclerotic lesion area was calculated using ORO staining from the 10 sections at 40 μm intervals, starting at the appearance of at least two aortic valves, as described previously^24^.

### Blood lipid analysis

Blood lipids were detected in plasma samples, collected for all four genotypes. Hemolytic samples were discarded. Blood lipid analyses included determination of total cholesterol, high-density lipoprotein cholesterol (HDLC), direct low-density lipoprotein cholesterol (LDLC), and triglycerides. Samples were analyzed using a Roche Cobas 702 instrument at the Institute for Clinical Chemistry of the University of Lübeck.

### Lipid profiling

#### Sample preparation

Plasma samples were extracted according to a modified version of the protocol of Pellegrino *et al*. using a mixture of methanol, methyl tertiary-butyl ether, and CHCl_3_ (1.33:1:1; v/v/v), to which butylated hydroxytoluene (100 mg/L, Sigma-Aldrich, Schnelldorf, Germany) and 2.5 µl of SPLASH internal standard mix (Avanti Polar Lipids, Alabaster, AL, USA) were added^25^. Samples were vortexed for 30 s, incubated at 25 °C on a shaker for 30 min, and centrifuged for 10 min at 2000 × g, and supernatants were collected and dried under vacuum. Subsequently, samples were resuspended in 50 µl of methanol/isopropyl alcohol (1:1, v/v), centrifuged for 10 min at 16,000 × g, and transferred into liquid chromatography mass spectrometry (LC-MS) vials.

#### LC-MS analysis

Analysis was performed on an Orbitrap instrument (QExactive, ThermoFisher Scientific, Bremen, Germany) coupled to a Dionex Ultimate 3000 LC system (ThermoFisher Scientific, Bremen, Germany). Separation was performed on an Accucore C30 RP column (150 × 2.1 mm, particle size: 2.6 µm) using AcN/H_2_O 6:4 (*v/v*), 0,1% FA as solvent A, and IPA/AcN (9:1, *v/v*), 0,1% FA as solvent B, flow was held at 0.26 ml/min. All solvents were of LC-MS grade quality and were purchased from Merck (Darmstadt, Germany). The following gradient was used: Starting at 30% B ramping to 43% B after 2 min, to 55% B at 2.1 min to 65% B at 12 min, 85% B at 18 min and 100% B after 20 min. Subsequently the columned was washed at 100% B for 15 min and re-equilibrated to starting conditions for 3 min^26^.

#### Data analysis

Data were recorded in positive and negative ion modes at resolutions of R = 70,000 for full scans and R = 17,500 for MS/MS scans. MS/MS data were generated using data-dependent acquisition. Data evaluation was performed using TraceFinder (ThermoFisher Scientific, Bremen, Germany). Lipid species were identified with an in-house library, according to exact mass, retention time, fragmentation ions, and isotopic pattern, using the same method as Karsai *et al*.^27^.

### Steroid measurement

Various steroids, including progesterone, testosterone, deoxycorticosterone, corticosterone, and aldosterone, as well as estrogens including estrone (E1), estradiol (E2) and estriol (E3) (Concentrations less than 10 pmol/L were considered as not detectable) were measured in mouse plasma samples, according to protocols established for human analysis. All hormones were determined by LC-MS/MS, as previously described^28,29^. Briefly, sample aliquots, calibrators, and controls were combined with the internal standard mixture to monitor recovery. All samples were extracted using Oasis MAX SPE system plates (Waters, Milford, MA, USA). Chromatographic separation was carried out using an UPLC system, connected to a Quattro Premier/XE triple Quad mass spectrometer (Waters, Milford, MA, USA). A Waters Acquity UPLC BEH C18 column (1.7 μm, 100 × 2.1) was used at a flow rate of 0.4 ml/min at 50 °C. Water (A) and acetonitrile (B) with 0.01% formic acid were used as the mobile phase. Two mass transitions were monitored for each hormone. The following optimized parameters were used: capillary voltage, 3.5 kV; cone voltage, 28–33 V; collision energy, 18–25 eV; dwell time, 0.01–0.08 s, depending on the steroid; source temperature, 120 °C; and desolvation temperature, 450 °C. Argon was used as the collision gas. Data were acquired using MassLynx 4.1 software and quantified with QuanLynx software (Waters, Milford, MA, USA). During all analyses, the ambient temperature was maintained at 21 °C by air conditioning.

### Statistical analysis

Data are presented as the mean ± standard error of the mean (SEM). For multiple comparisons, one- or two-way ANOVA was used, as appropriate, followed by recommended post-hoc tests. For comparisons of two groups, unpaired t-tests were performed. Analyses were performed using GraphPad Version 6.0d (GraphPad Software Inc., San Diego, CA). *P-*values <0.05 was considered statistically significant.

## Results

### *Cyp17a1* KO mice had increased body weight and visceral/subcutaneous fat deposition, altered steroid metabolism, and altered lipid metabolism

After the generation of *Cyp17a1* KO mice and confirmation of KO by PCR analysis at genomic DNA level (Fig. 1a,b**)**, qPCR analysis at RNA level (Supplemental Fig. 2d) and immunhistostaining at protein level (Supplemental Figs. 1c,d and 2a,b), mice were initially characterized in our laboratory at 4–6 months old. XY KO mice had a female appearance (external genital phenotype) **(**Fig. 1c**)**; however, genotyping revealed a Mendelian ratio of XX and XY chromosomes (1:1), resembling the human phenotype in the rare form of the DSD and CAH, caused by CYP17A1 deficiency^30^. Evaluation of various steroid hormones in plasma from KO mice revealed lack of testosterone synthesis and significantly elevated corticosterone and desoxycorticosterone levels in XY mice **(**Table 1**)**. In XX mice, a marked accumulation of progesterone and a minor elevation of corticosterone levels were observed **(**Table 1**)**.

**Table 1:** Hormonal measurement in WT and KO mice in plasma levels by LC-MS/MS.

**Hormones (in nmol/l)**	**XX mice**		***P-*value**	**XY mice**		***P-*value**
	***Cyp17A*** (+/+) **(n** = **9) (mean** ± **SEM)**	***Cyp17A**** (d/d)* **(n** = **6) (mean** ± **SEM)**		***Cyp17A*** (+/+) **(n** = **6) (mean** ± **SEM)**	***Cyp17A**** (d/d)* **(n** = **12) (mean** ± **SEM)**	
Progesterone	8.53 ± 5.59	37.61 ± 0.9.88	**0.001**	1.08 ± 0.21	2.36 ± 1.53	0.067
Desoxycorticosterone	8.47 ± 3.50	12.69 ± 2.89	0.216	2.03 ± 0.64	11.60 ± 7.49	**0.008**
Corticosterone	405.55 ± 81.94	617.35 ± 137.48	0.128	254.47 ± 42.62	699.43 ± 313.30	**0.002**
Aldosterone	0.42 ± 0.13	0.57 ± 0.15	0.382	0.41 ± 0.12	1.01 ± 0.94	0.154
Testosterone	0.12 ± 0.05	0.10 ± 0.03	0.770	11.53 ± 6.22	0.04 ± 0.03	**0.015**
Estrone	0.11 ± 0.02	0.0 ± 0.0	**0.001**	—	—	—

In general, WT male mice have higher body weights than females. C*yp17a1* KO XX mice exhibited a marked increase in body weight, reaching values typical for WT males and significantly higher than those of WT females **(**Fig. 1d, Table 2). In-depth characterization revealed higher visceral/subcutaneous fat deposition in XX KO mice **(**Fig. 1e**)**.

**Table 2:** Body weight and various lipid parameter levels measurement in WT and KO.

	**XX mice**		***P-*value**	**XY mice**		***P-*value**
	***Cyp17A*** (+/+) **(n** = **7) (mean** ± **SEM)**	***Cyp17A**** (d/d)* **(n** = **6) (mean** ± **SEM)**		***Cyp17A*** (+/+) **(n** = **6) (mean** ± **SEM)**	***Cyp17A**** (d/d)* **(n** = **12) (mean** ± **SEM)**	
Body weight (in g)	22.40 ± 0.75	26.20 ± 0.54	**0.001**	32.25 ± 0.43	34.15 ± 1.14	0.269
HDLC (mmol/l)	1.01 ± 0.09	1.06 ± 0.06	0.652	1.27 ± 0.13	1.32 ± 0.05	0.700
LDLC (mmol/l)	0.19 ± 0.01	0.15 ± 0.01	**0.023**	0.11 ± 0.02	0.19 ± 0.01	**0.003**
Total Cholesterol (mmol/l)	2.29 ± 0.20	2.31 ± 0.13	0.947	2.43 ± 0.29	3.17 ± 0.15	**0.039**
Triglycerides (mmol/l)	0.73 ± 0.04	0.75 ± 0.03	0.666	0.84 ± 0.09	0.80 ± 0.03	0.588

Significant changes in LDLC levels were observed in both XY (increase) and XX (decrease) KO animals compared to WT (Table 2). We also observed a significant increase of total cholesterol in XY KO mice. No significant differences were noted in, HDLC or triglycerides in females of different genotypes. In XY animals, there was a significant difference in total cholesterol between KO and WT animals (Table 2).

#### Cyp17a1 expression

*Cyp17a1* KO mice were generated by insertion of a targeting vector containing a *lacZ* gene between exon 3 and 4 of *Cyp17a1* (Fig. 1a). Insertion of lacZ into the *Cyp17a1* gene allows monitoring of tissue-specific *Cyp17a1* expression by X-gal staining. As expected, fertile heterozygous male mice show a strong tissue-specific expression of lacZ in the Leydig cells (Supplemental Fig. 1c**)**. In addition a staining of the epithelium of the epididymis was observed **(**Supplemental Fig. 1d**)**. This tissue-specific expression was confirmed by CYP17A1 staining of testis from heterozygous embryos and WT males using polyclonal antibody sc-46081 (Santa Cruz Biotechnology) (Supplemental Fig. 1a,b,e).

Furthermore, we also used immunohistological staining to determine the expression of CYP17A1 in aortic plaques of an old Apoe KO mouse with atherosclerotic deposits. Few infiltrated cells were found to express CYP17A1 at very low level **(**Supplemental Fig. 2c).

To test the expression of CYP17A1 in human materials we conducted gene expression analysis in different cell lines that play a role during the initiation and development of atherosclerosis such as monocytes, aortic smooth muscle cells (HASMCs) and endothelial cells (HUVECS). At basal conditions, none of those cells express CYP17A1 at RNA level. For HUVECS data are not shown. Similar to the mouse finding we noticed that monocyte-derived macrophages and synthetic confluent HASMCs express CYP17A1 at very low level (Supplemental Fig. 3). Further, Genotype-Tissue Expression (GTEx) datastet (v8) confirmed our expression results^31^.

### Analysis of atherosclerosis

To study atherosclerosis in CYP17A1-deficient mice, animals were backcrossed onto the atherogenic *Apoe(d/d)* background. At 10 weeks old, mice were divided into four groups (female and male of each of *Cyp17a1(d/d)* x *Apoe(d/d)* and *Cyp17a1(*+/+) x *Apoe(d/d)* and fed either a WTD or a standard chow diet for a further 8 weeks. Various parameters associated with atherosclerosis, including body weight, visceral fat deposits, and lipid parameters (total cholesterol, LDLC, HDLC, and triglycerides), were assessed in the mice, and atherosclerotic deposits in the entire aorta and the aortic root were evaluated.

#### CYP17A1-deficient mice on the Apoe(d/d) background exhibited increased body weight and visceral/subcutaneous fat deposition in XX females lacking Estrone

**Figure 2:**
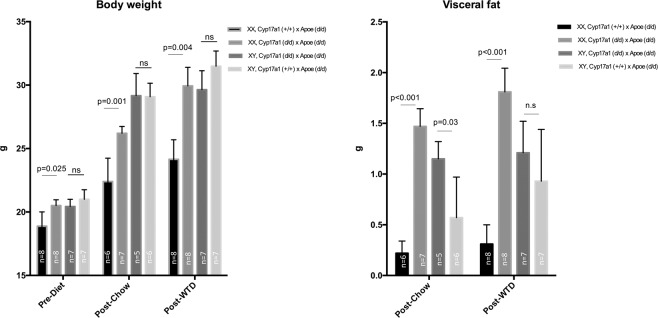
Body weight and visceral fat in *Cyp17a1* KO and WT mice on an *Apoe* KO atherogenic background. Body weight and visceral fat were assessed in mice at 10 weeks old, before dietary differentiation, and after 8 weeks consuming a WTD or a standard chow diet. ns, not significant.

Similar to the observations in mice on a non-atherogenic background, increased body weight was observed only in *Cyp17a1(d/d)* x *Apoe(d/d)* XX mice consuming either standard chow or WTD, relative to the *Apoe(d/d)* controls **(**Fig. 2**)**. No significant differences in body weight were observed between XY KO and XY WT. Only a slight increase in body weight was observed in each group attributable to the WTD. Furthermore, a dramatic increase in visceral fat deposition was observed in XX KO from the initiation of differential feeding to the end of the experiment in animals consuming both standard chow (mean ± SD: 0.22 ± 0.12 vs. 1.47 ± 0.46 g) and WTD (0.31 ± 0.19 vs. 1.81 ± 0.66 g) **(**Fig. 2**)**. In XY KO mice a significant increase in visceral fat was only observed in mice feeding the chow diet.

We also determined the level of estrogen parameters including Estrone (E1), Estradiol (E2) and Estriol (E3) in XX mice in each group of *Cyp17a1(d/d)* x *Apoe(d/d)* and *Cyp17a1(*+/+) x *Apoe(d/d)*) under chow diet. Due to the low sensitivity of the assay, we were only able to detect measurable Estrone (E1). The Cyp17a1 KO mice lack Estrone compared to WT (Table 1).

#### Lipid parameters in CYP17A1-deficient mice on the Apoe(d/d) atherogenic background

As shown in Table 3, mice fed with a WTD for 8 weeks exhibited demonstrable increases in total cholesterol and LDLC, as expected; however, in contrast to mice on a non-atherogenic background, no significant differences were observed between the genotypes in total cholesterol or LDLC in animals of either chromosomal sex. Also, no differences in triglycerides were detected between mice with different genotypes. Only a slight but significant difference in TC was observed in XX Cyp17a1 (d/d) x Apoe (d/d) compared to Cyp17a1 (+/+) x Apoe (d/d) at the age of 8 weeks before starting with WTD diet (Table 3).

**Table 3:** Lipid parameter levels measurement in WT and KO on Apoe (d/d) genetic background after feeding a WTD.

Lipid parameter	Status	XX mice		*P-*value	XY mice		*P-*value
		Cyp17a1 (+/+) x Apoe (d/d) (n = 7) (mean ± SEM)	Cyp17a1 (d/d) x Apoe (d/d) (n = 7) (mean ± SEM)		Cyp17a1 (d/d) x Apoe (d/d) (n = 7) (mean ± SEM)	Cyp17a1 (+/+) x Apoe (d/d) (n = 6) (mean ± SEM)	
TC	Pre-Diet	6.59 ± 0.30	7.56 ± 0.19	**<0.001**	7.68 ± 0.48	7.72 ± 0.51	0.961
	Post-Chow	7.08 ± 0.76	7.02 ± 0.40	0.944	9.48 ± 1.24	8.84 ± 1.08	0.718
	Post-WTD	20.83 ± 0.92	22.24 ± 1.27	0.433	22.14 ± 1.45	19.61 ± 2.40	0.372
LDLC	Pre-Diet	3.3 ± 0.28	4.03 ± 0.25	0.062	3.96 ± 0.38	3.78 ± 0.36	0.734
	Post-Chow	3.7 ± 0.29	4.27 ± 0.29	0.202	4.64 ± 0.55	5.88 ± 0.18	0.063
	Post-WTD	9.73 ± 0.83	10.41 ± 0.70	0.540	11.00 ± 0.55	10.77 ± 1.06	0.845
HDLC	Pre-Diet	0.69 ± 0.04	0.63 ± 0.04	0.225	0.68 ± 0.03	0.68 ± 0.04	0.963
	Post-Chow	0.77 ± 0.44	0.44 ± 0.04	0.432	3.25 ± 1.23	0.55 ± 0.09	0.062
	Post-WTD	0.28 ± 0.04	0.37 ± 0.05	0.149	0.47 ± 0.09	0.45 ± 0.06	0.867
TG	Pre-Diet	1.3 ± 0.24	1.43 ± 0.11	0.576	1.41 ± 0.12	1.38 ± 0.14	0.855
	Post-Chow	1.46 ± 0.24	1.16 ± 0.05	0.222	2.77 ± 0.51	1.90 ± 0.18	0.143
	Post-WTD	2.1 ± 0.16	2.11 ± 0.24	0.950	2.55 ± 0.89	2.18 ± 0.42	0.262

#### CYP17A1-deficiency on an Apoe KO genetic background leads to increased atherosclerosis in XY but not in XX mice

**Figure 3:**
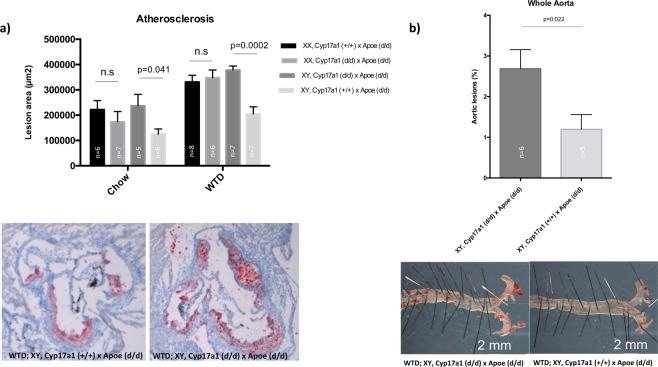
Atherosclerosis analyses in mice fed chow or WTD. (**a**) Quantification of plaque deposits in aortic roots and representative images after Oil Red O staining (red). (**b**) Oil Red O quantification and staining of atherosclerotic lesions in the whole aorta of WTD fed mice. ns, not significant.

First, we assessed atherosclerotic plaques in the aortic root. As expected, we detected an increase in atherosclerotic deposits after consumption of the WTD compared with standard chow in all groups (Fig. 3a). Analysis of the lesion area in aortic roots from XY mice lacking CYP17A1 revealed a large number of vascular lesions in those consuming both the WTD and chow diet, relative to controls **(**Fig. 3a**)**. No difference in atherosclerotic deposits was detected between XX mice of different genotypes consuming either chow or WTD. Similarly, remarkably significant differences in atherosclerotic deposits were also detected by en-face quantification of the whole aorta between XY mice in the *Cyp17a1* KO and WT groups **(**Fig. 3b**)**.

#### Lipid metabolic profiling

**Figure 4:**
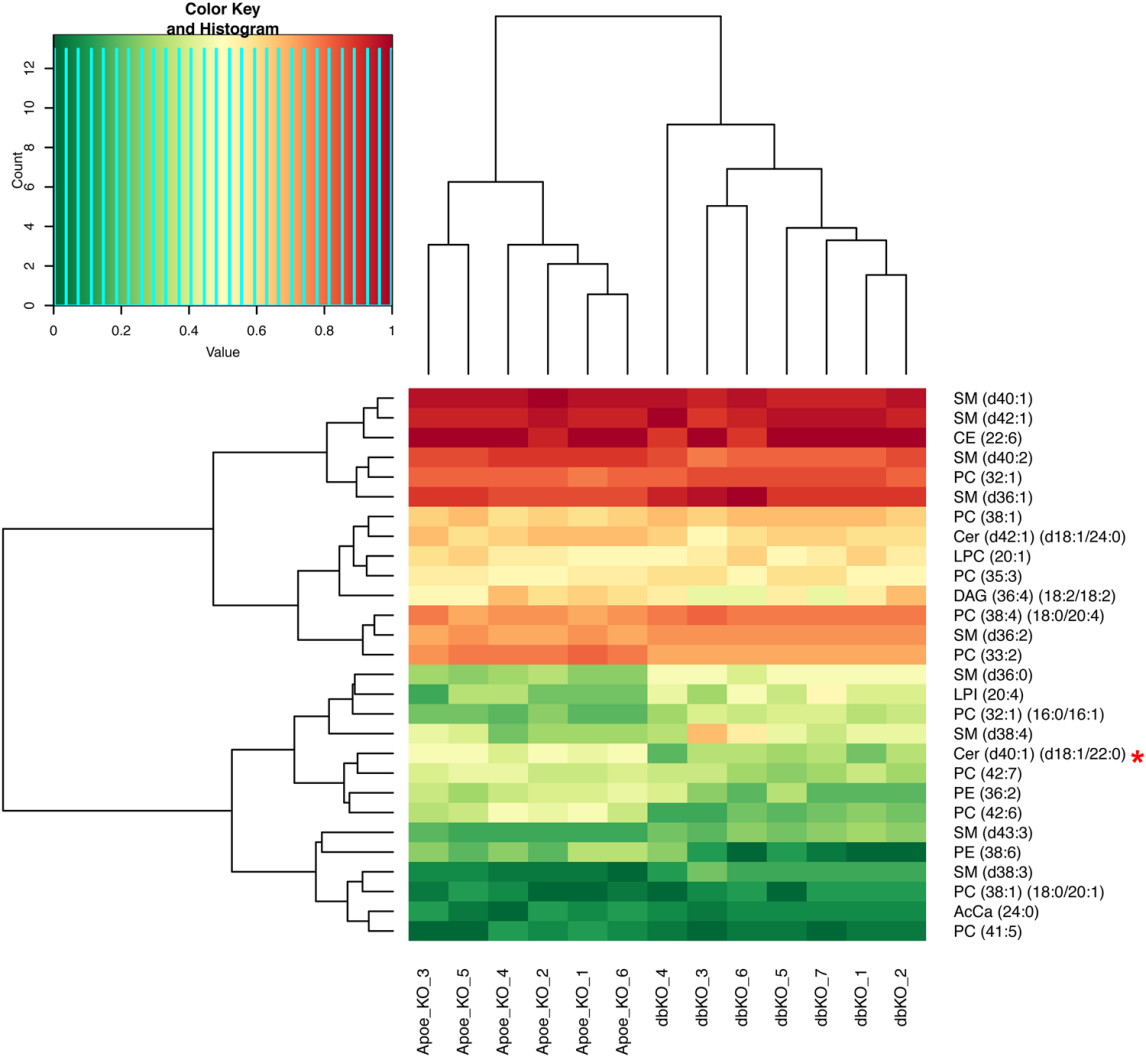
Heatmap representing lipid species showing differences in levels between XY mice with a *Cyp17a1 (d/d)* x *Apoe (d/d)* (dbKO) and *Cyp17a1* (+/+) x *Apoe (d/d)* (Apoe_KO) genotype. Lipids were extracted from plasma samples and evaluated by LC-MS. The levels of each lipid species were set from 0 to 1. The heatmap shows the difference in levels of lipid species between CYP17A1 deficient double KO mice (dbKO) and controls (*Apoe*_KO) mice. The asterisk (*) indicates lipid species with significantly different levels after correction for multiple testing (Bonferroni-corrected *P*-value threshold of 2.1 × 10^−4^ (0.05/234 all lipid species)).

Atherosclerosis is believed to be caused by disorders of lipid metabolites. Therefore, we focused on XY mice and conducted mass spectrometry analysis to profile 234 lipid species in plasma samples. Twenty-eight lipid species showed distinct profiles between *Cyp17a1(d/d)* x *Apoe(d/d)* and *Cyp17a1(*+/+) x *Apoe(d/d)* mice. After using Bonferroni-corrected *P*-value threshold of 2.1 × 10^−4^ (0.05/234 all lipid species), only the ceramide species, Cer (d40:1) (d18:1/22:00), differed significantly between the two groups **(**Fig. 4**)**.

## Discussion

CYP enzymes have crucial roles in steroid hormone synthesis; hence any genetic variation affecting them influences the balance of hormone synthesis, leading to various DSDs. Such disorders are also associated with various cardiovascular risk factors, including hypertension^32–37^, diabetes, and aortic stiffness^38^. Here we focused on *CYP17A1*, a gene mapping to a locus associated with various cardiovascular disorders, including CAD, MI, diabetes, and arterial stiffness, according to GWAS^3,33,34,38–40^.

We knocked out the *Cyp17a1* gene in mice and characterized various parameters in KO animals. In contrast to a previous publication reporting embryonic lethality in CYP17A1-deficient mice^41^, our mice survived to adulthood. This is corroborated by the fact that in humans, severe mutations of CYP17A1 cause complete 17α-hydroxylase/17,20-lyase deficiency (17OHD), leading to disrupted steroidogenesis in adrenals and gonads and consequently to a rare form of DSD, but are compatible with life. We confirmed the expected steroid synthesis imbalance in our mice and observed a sex development disorder, resembling the human phenotype. XY KO mice lacked testosterone and appeared phenotypically females. The 17,20 lyase activity of CYP17A1 as well as the availability of 17-hydroxysteroid substrates, which are exclusive products of CYP17A1 are essential for the production of androgens and estrogens^30^. Like humans with severe 17OHD, mice deficient in CYP17A1 are infertile. Male *Cyp17a1* KO mice have small testis and lacking Wolffian derivates and female *Cyp17a1* KO mice display underdeveloped uteri **(**Supplemental Fig. 4**)**. Spontaneous fertility in woman with CYP17A1 deficiency has not been reported^42^, but a successful live birth after *in vitro* fertilization, embryo transfer and adequate endometrial preparation had been achieved^43^. Therefore, this mouse model reflects in many aspects the DSD phenotype of human 17OHD.

Here, we were interested in the impact of lack of testosterone on atherosclerosis and related risk factors, such as lipid parameters. CAD is a complex disease involving numerous modifiable and un-modifiable risk factors^1,44,45^. Men are more susceptible to, and likely to die of CAD than women of a similar age. The male-to-female ratio of mortality from vascular disease is approximately 3:1 worldwide^46^. Previous studies indicate that low level or absent testosterone is associated with an increase in CAD events^18–20,47,48^, and testosterone declines with obesity and age^47^. An increase in atherosclerotic plaque deposition was detected in XY mice lacking CYP17A1, and consequently lacking testosterone, suggesting a protective role for this hormone against atherosclerotic plaque formation. This is consistent with the recent report from Steinfeld and co-workers^49^, who demonstrated low plasma levels of testosterone in the *Apoe* and *Ldlr* mouse KO models for atherosclerosis, compared with non-atherogenic wild-type C57BL/6 controls in age-matched groups, receiving a standard chow diet. Further, our data are in line with those from previous studies of the effects of reduced testosterone levels in males castrated due to prostate cancer and in experimental mice^50,51^; in both cases, low testosterone led to increased atherosclerosis. This suggests that the effect of CYP17A1 deficiency on atherosclerosis in males seems to be acting via lack of testosterone because *Cyp17a1* is not or low expressed in cells playing a critical role in atherosclerosis including monocytes/macrophages, smooth muscle cells and endothelial cells, as well as the plaque itself. Thus physiological testosterone replacement could be an option to rescue the phenotype in mice^50^ without any need of genetic modification of *Cyp17a1* expression. Further these data support the GWAS finding that the chromosomal region containing the *CYP17A1* gene is associated with CAD and pinpoint *CYP17A1* as the causal gene at this locus^2,39,40^.

We initially found that absence of CYP17A1 was associated with elevated progesterone in XX KO mice. In addition, XX *Cyp17a1* KO mice displayed increased body weight and a massive accumulation of visceral fat deposits. Many studies have reported that women exhibit decreased progesterone, along with increases in cholesterol and body weight, after menopause^52–54^, and these factors increase the risk for CAD^55^. Surprisingly, no changes in atherosclerosis development were detected in XX *Cyp17a1* KO mice, although previous studies reported enhanced atherosclerosis in castrated females^56–58^. This may suggest that high level of progesterone in XX *Cyp17a1* KO mice may play an atherprotective role.

LDLC was the only factor observed to be decreased in both chromosomal sexes as a result of knocking out *Cyp17a1*. Low testosterone and estrogen parameters (E1–3) levels are predictive for the development of the metabolic syndrome and its consequence on CVD in men and women, respectively. This study aimed to investigate the influence of testosterone deficiency on atherosclerosis in males. However and due to the interesting obesity phenotype in XX *Cyp17a1* KO, we tried to determine the level of E1–3 in remaining plasma material in XX *Cyp17a1* KO mice on an *Apoe* genetic background and under chow diet. The analysis revealed a complete absence of Estrone (E1) in XX *Cyp17a1* KO mice as compared to the WT and are in line with previous work on ovariectomiszd females^59^. We were not able to detect E2–3 in our samples even in WT more likely due to the assay sensitivity limitation. The dysregulation of estrogen metabolism has also been associated with the susceptibility to obesity. Menopause associated reductions in estrogen are linked to a significant increase in the incidence of obesity^53^. Exogenous estrogen attenuates ovariectomy induced weight gain in mouse models^60^. Future studies are required to study the effect of Estradiol supplementation on obesity in our mouse model.

To understand the mechanisms underlying the association between low testosterone and atherosclerosis, we tested the effects of CYP17A1-deficiency on lipid metabolism, as a plausible contributor to atherosclerosis in our mouse model. We analyzed mice on both non-atherogenic and atherogenic *Apoe* backgrounds, fed with either standard chow or a WTD. We observed alterations of both total and LDLC levels after knocking out *Cyp17a1*, particularly in non-atherogenic males; however, no big differences were observed in mice on the atherogenic background. This may be due to the boost in cholesterol driven by consumption of the WTD; however, lipidomic profiling only demonstrated significant changes in one specific ceramide species Cer (d40:1) (d18:1/22:00), using Bonferroni-corrected *P*-value threshold of 2.1 × 10^–4^ (0.05/234 all lipid species) between *Cyp17a1(d/d)* x *Apoe(d/d)* XY mice and *Apoe(d/d)* littermate controls fed the WTD. The role of ceramides in atherosclerosis has been reported previously^61^. Together with fatty acids, ceramide forms the backbone of sphingolipids, which are key to the initiation and development of atherosclerosis^61^. Ichi *et al*. previously reported an association of ceramides in human plasma with risk factors for atherosclerosis^62^. The exact role of Cer (d40:1) (d18:1/22:00) remains to be determined in future studies.

In this study, we generated a *Cyp17a1* KO mouse model of the rare form of the human DSD, caused by mutations in *CYP17A1*. The KO mice were non-fertile, due to a defect in sex hormone biosynthesis, and exhibited higher corticosterone levels in XY mice. It is conceivable that corticosterone release was stimulated by inflammatory cytokines and nutritional overflow which in turn accelerates the development of atherosclerotic changes in double *Cyp17a1-ApoE* double-knockout XY mice. Corticosterone accelerates atherosclerosis in the apolipoprotein E-deficient mouse^63^.

In addition our KO mice were not embryonic lethal. Here we investigated the effects of low testosterone in males on atherosclerosis and lipid metabolism in more details. Our data demonstrate that CYP17A1 influences lipid levels, likely contributing to the role of this factor in influencing CAD risk.

## Supplementary information

Supplementary Figure 1.

Supplementary Figure 2.

Supplementary Figure 3.

Supplementary Figure 4.
