# Supplementary material for: CYP17A1 deficient XY mice display susceptibility to atherosclerosis, altered lipidomic profile and atypical sex development
Source: Sci Rep. 2020 May 29;10:8792. doi: 10.1038/s41598-020-65601-0 (PMC7260244; doi:10.1038/s41598-020-65601-0)
Supplement: Supplementary file 1 — Supplementary Figure 1. [file 41598_2020_65601_MOESM1_ESM.pdf]

CYP17A1 deficient XY mice display susceptibility to atherosclerosis, altered lipidomic profile and atypical sex development

Redouane Aherrahou[a,b], Alexandra E. Kulle[c], Natalia Alenina[d,e], Ralf Werner[f,g], Simeon Vens-Cappell[h] , Michael Bader[d,e,i,j], Heribert Schunkert[k], Jeanette Erdmann[a,l, *], Zouhair Aherrahou[a,l, *]

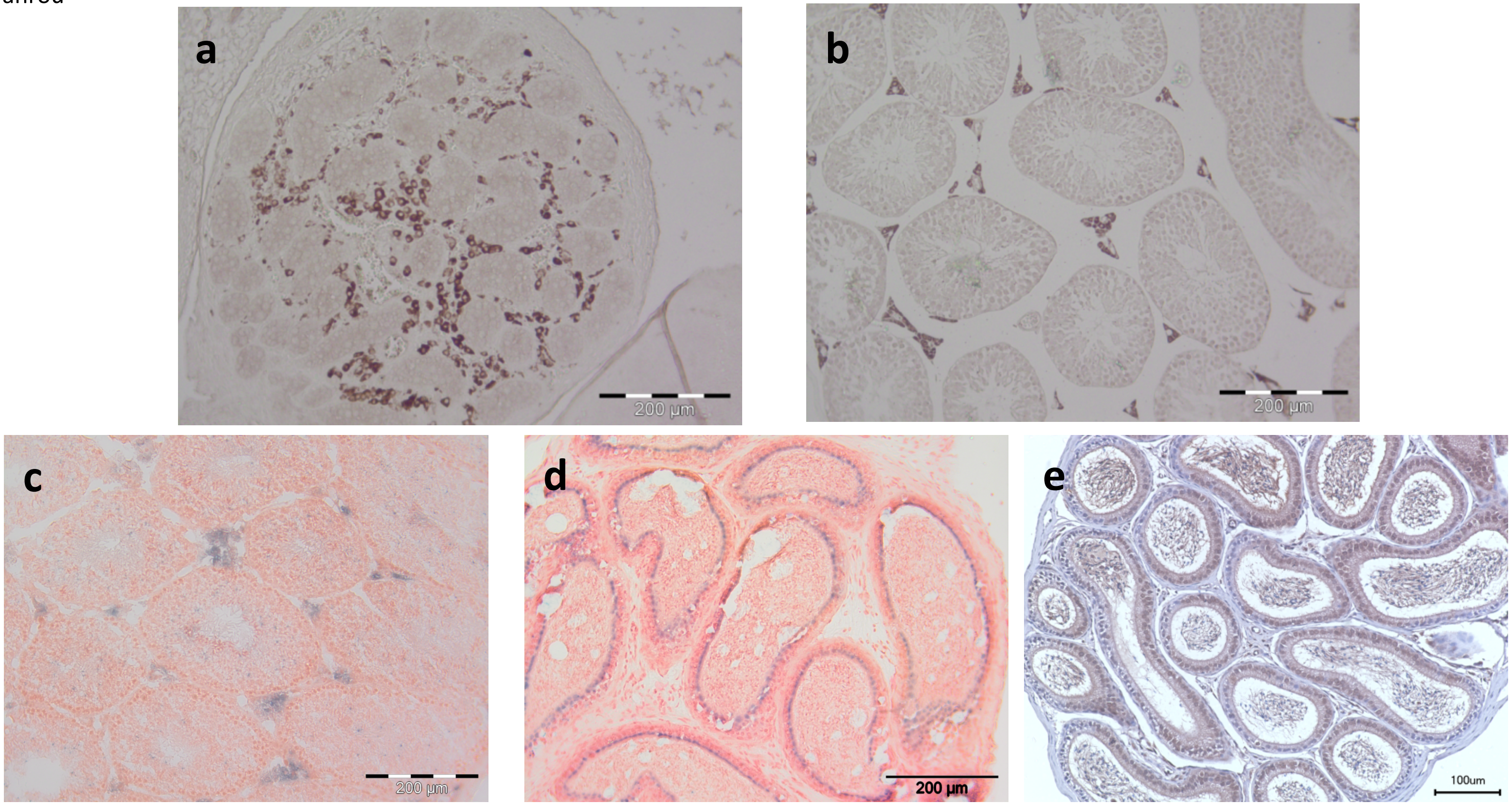

Supplementary Figure 1: Protein expression of CYP17A1. Immunhistological staining (brown) revealed the expression of CYP17A1 in testis of an E16 heterozygous *Cyp17a1* (+/d) mouse embryo in Leydig cells (A). CYP17A1 is also expressed in Leydig cells of a WT adult mouse testis (B). Similarly the expression of CYP17A1 was also demonstrated in Leydig cells in the interstitium (C) and in the epithelium of the epididymis (D) using X-gal staining (blue). The Expression of CYP17A1 found in the epididymis using X-gal staining, was further validated in the epididymis using immunhistological staining (E, brown).
