# Supplementary material for: CYP17A1 deficient XY mice display susceptibility to atherosclerosis, altered lipidomic profile and atypical sex development
Source: Sci Rep. 2020 May 29;10:8792. doi: 10.1038/s41598-020-65601-0 (PMC7260244; doi:10.1038/s41598-020-65601-0)
Supplement: Supplementary file 2 — Supplementary Figure 2. [file 41598_2020_65601_MOESM2_ESM.pdf]

CYP17A1 deficient XY mice display susceptibility to atherosclerosis, altered lipidomic profile and atypical sex development

Redouane Aherrahrou[a,b], Alexandra E. Kulle[c], Natalia Alenina[d,e], Ralf Werner[f,g], Simeon Vens-Cappell[h] , Michael Bader[d,e,i,j], Heribert Schunkert[k], Jeanette Erdmann[a,l, *], Zouhair Aherrahrou[a,l, *]

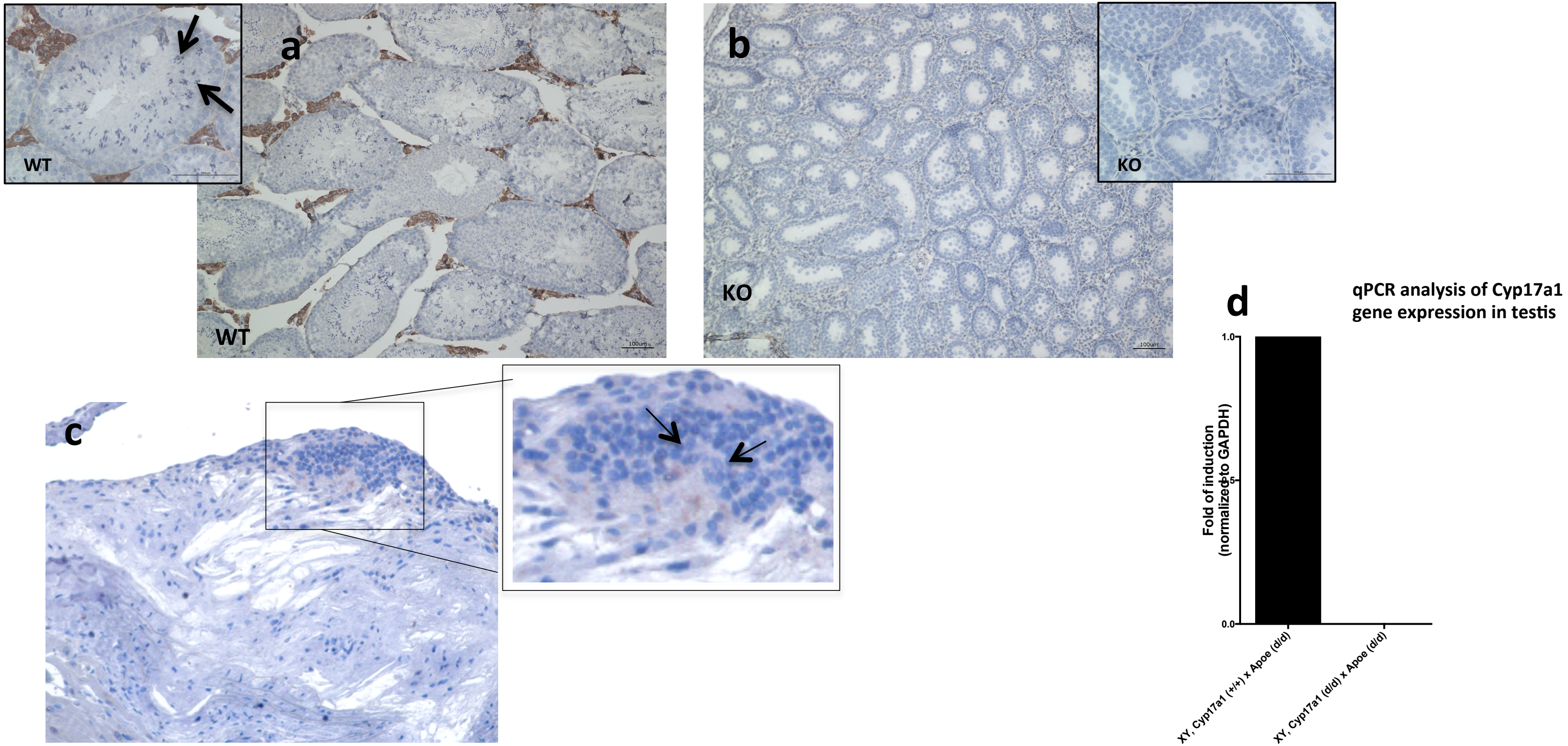

Supplementary Figure 2: Expression of CYP17A1. A: Testis of an adult *Cyp17a1* (+/+)xApoe(d/d) mouse showing normal seminiferous tubules and spermatogenesis processes (arrays, high magnification) as well as the expression of Cyp17a1 in Leydig cells using immunhistological staining (brown). B: Testis of an aged-matched Cyp17a1(d/d)xApoe(d/d) mouse showing smaller tubules, missing spermatogenesis, and no expression of CYP17A1. C: Expression of CYP17A1 in aortic plaque cap of an old Apoe(d/d) mouse (1 year old). Cell nuclei were stained blue using Heamalaun counterstaining. D: qPCR analysis of the expression of Cyp17a1 in testis of adult adult *Cyp17a1* (+/+)xApoe(d/d) mouse. All bar scales are set to 100 um.
