# Supplementary material for: CYP17A1 deficient XY mice display susceptibility to atherosclerosis, altered lipidomic profile and atypical sex development
Source: Sci Rep. 2020 May 29;10:8792. doi: 10.1038/s41598-020-65601-0 (PMC7260244; doi:10.1038/s41598-020-65601-0)
Supplement: Supplementary file 3 — Supplementary Figure 3. [file 41598_2020_65601_MOESM3_ESM.pdf]

# CYP17A1 deficient XY mice display susceptibility to atherosclerosis, altered lipidomic profile and atypical sex development

Redouane Aherrahrou[a,b], Alexandra E. Kulle[c], Natalia Alenina[d,e], Ralf Werner[f,g], Simeon Vens-Cappell[h] , Michael Bader[d,e,i,j], Heribert Schunkert[k], Jeanette Erdmann[a,l, *], Zouhair Aherrahrou[a,l, *]

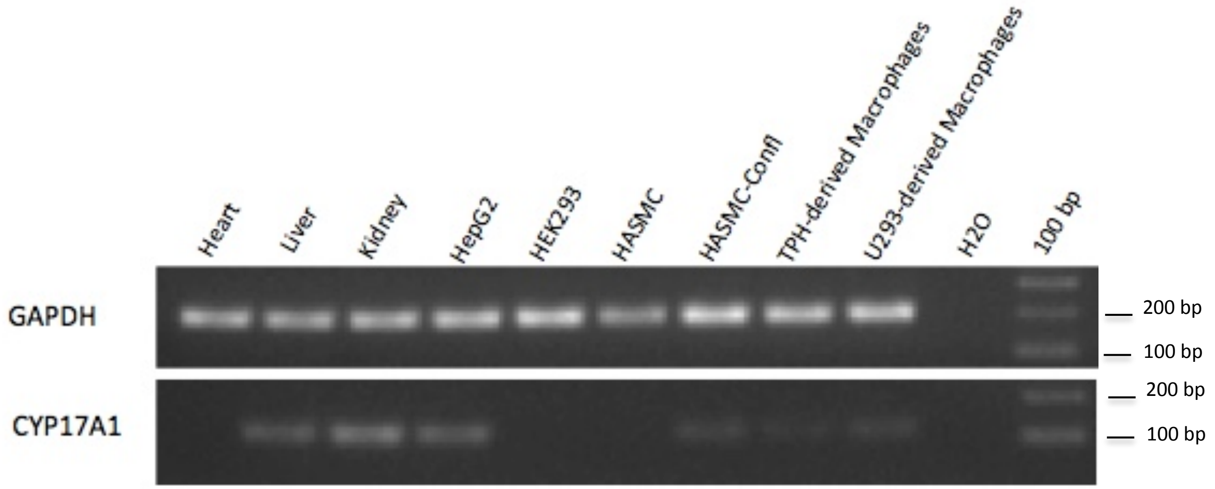


**Supplementary Figure 3: qPCR analysis of CYP17A1 in different human atherosclerotic cell lines and organ*s:*** Gene expression analysis of CYP17A1 was performed in various human tissues: Heart, Liver, Kidney, and cells types: liver cells (HepG2), human embryonic kidney cells (HEK293), Human aortic smooth muscle cells (HASMC), confluent HASMC, TPH-1 and U293 monocytes-derived macrophages cells.
