# Supplementary material for: CYP17A1 deficient XY mice display susceptibility to atherosclerosis, altered lipidomic profile and atypical sex development
Source: Sci Rep. 2020 May 29;10:8792. doi: 10.1038/s41598-020-65601-0 (PMC7260244; doi:10.1038/s41598-020-65601-0)
Supplement: Supplementary file 4 — Supplementary Figure 4. [file 41598_2020_65601_MOESM4_ESM.pdf]

CYP17A1 deficient XY mice display susceptibility to atherosclerosis, altered lipidomic profile and atypical sex development

Redouane Aherrahrou[a,b], Alexandra E. Kulle[c], Natalia Alenina[d,e], Ralf Werner[f,g], Simeon Vens-Cappell[h] , Michael Bader[d,e,i,j], Heribert Schunkert[k], Jeanette Erdmann[a,l, *], Zouhair Aherrahrou[a,l, *]

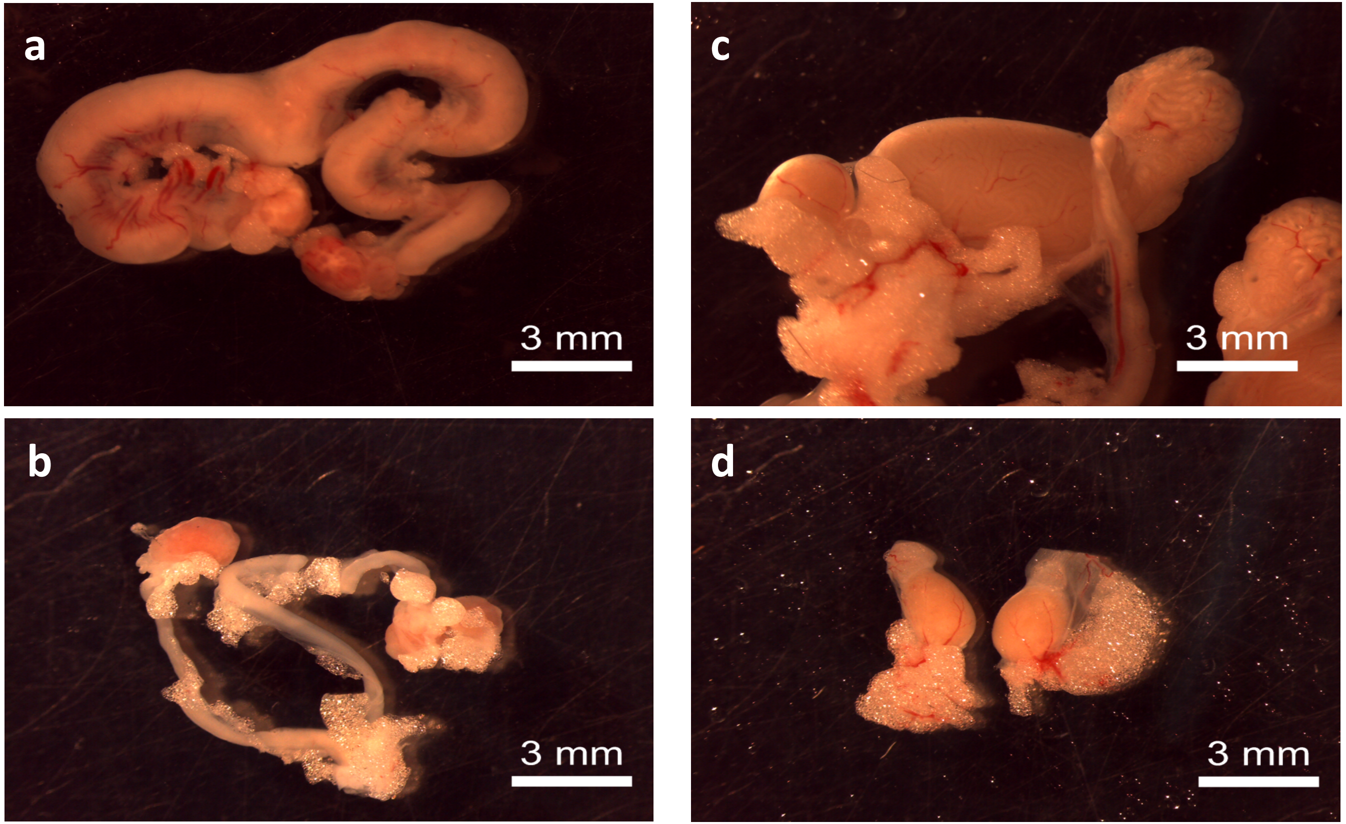

Supplementary Figure 4: Comparison of inner genitalia of six month old mice. (A) Uteri and ovaries of a WT mouse. (B) Uteri and ovaries of a *Cyp17a1* KO mouse. (C) Testis of a WT mouse. (D) Testes of a *Cyp17a1* KO mouse.
